# Privacy-preserving approximate GWAS computation based on homomorphic encryption

**DOI:** 10.1186/s12920-020-0722-1

**Published:** 2020-07-21

**Authors:** Duhyeong Kim, Yongha Son, Dongwoo Kim, Andrey Kim, Seungwan Hong, Jung Hee Cheon

**Affiliations:** Department of Mathematical Sciences, Seoul National University, 1, Gwanak-ro, Gwanak-gu, Seoul, Republic of Korea

**Keywords:** Homomorphic encryption, Privacy, GWAS, Fisher scoring

## Abstract

**Background:**

One of three tasks in a secure genome analysis competition called iDASH 2018 was to develop a solution for privacy-preserving GWAS computation based on homomorphic encryption. The scenario is that a data holder encrypts a number of individual records, each of which consists of several phenotype and genotype data, and provide the encrypted data to an untrusted server. Then, the server performs a GWAS algorithm based on homomorphic encryption without the decryption key and outputs the result in encrypted state so that there is no information leakage on the sensitive data to the server.

**Methods:**

We develop a privacy-preserving semi-parallel GWAS algorithm by applying an approximate homomorphic encryption scheme HEAAN. Fisher scoring and semi-parallel GWAS algorithms are modified to be efficiently computed over homomorphically encrypted data with several optimization methodologies; substitute matrix inversion by an adjoint matrix, avoid computing a superfluous matrix of super-large size, and transform the algorithm into an approximate version.

**Results:**

Our modified semi-parallel GWAS algorithm based on homomorphic encryption which achieves 128-bit security takes 30–40 minutes for 245 samples containing 10,000–15,000 SNPs. Compared to the true *p*-value from the original semi-parallel GWAS algorithm, the *F*_1_ score of our *p*-value result is over 0.99.

**Conclusions:**

Privacy-preserving semi-parallel GWAS computation can be efficiently done based on homomorphic encryption with sufficiently high accuracy compared to the semi-parallel GWAS computation in unencrypted state.

## Background

After the successful completion of the Human Genome Project in the early 21st century, high throughput technology on genetic variations has been rapidly developed and widely studied. In particular, through the development of microarray chip with rather small computational cost, it became possible to determine the genotype of millions of single nucleotide polymorphism (SNP), a variation in a single nucleotide that occurs at a specific position in the genome, for each individual. With those statistical data of genotypes, many researches are proposed that investigate associations between SNPs and phenotypes like major human disease, and especially Genome-wide association study (GWAS) aims to find top significant SNPs relevant to a certain phenotype.

### Motivation

Since genome analysis uses genomic data that are very sensitive and irreplacable, privacy on genomic data has come up to be one of the most important issues in genome analysis including GWAS. The usual privacy-preserving methodology in data analysis is anonymization, perturbation, randomization, and condensation [1]; however, those methods leverage the quality of data with privacy resulting in an inaccurate analysis to some extent. The dilemma regarding the balance between personal privacy and analytical efficiency has been resolved by applying many cryptographic primitives, while homomorphic encryption (HE) is noticed as one of the ultimate cryptographic solutions for privacy-preserving data analysis. Conceptually, HE is an encryption scheme which allows computations over encrypted data without decryption. HE not only fundamentally prevents the leakage of input data during the analysis phase, but also provides an accurate result of analysis since it preserves the original data intactly. However, HE causes a significant blowup of computational cost for analysis, and optimization and modification of algorithm for efficient computation in HE is the main problem of applying HE in data analysis.

Since 2014, there has been an annual biomedical privacy competition hosted by Integrating Data for Analysis, Anonymization and SHaring (iDASH), a national center for biomedical computing in the United States. One of three tasks in iDASH 2018 [2] was to develop a solution for privacy-preserving GWAS computation based on HE, and we participated in this competition with our delicately constructed algorithms.

### Summary of results

In this study, we propose approximate HE algorithms for privacy-preserving GWAS computation. To be precise, we transform well-known Fisher scoring and semi-parallel GWAS algorithm into *HE-friendly* algorithms so that we can efficiently evaluate them in encrypted state. Note that the HE-friendly modified Fisher Scoring algorithm can be generally used for logistic regression, not only for GWAS.

The main challenges in transforming the semi-parallel GWAS algorithm (Algorithm 1) to an HE algorithm are complex matrix operations such as multiplication and inversion. Since matrix inversion in HE is complicated and costly, we substitute it by computation of the adjoint matrices and determinant. With this approach, the original Fisher scoring algorithm can be successfully modified to compute encrypted data efficiently. For the efficient computation of semi-parallel GWAS computation based on HE, moreover, we reduced the number of matrix multiplications as many as possible, and modified the original algorithm into an approximate version which requires much less computational cost. The details of our optimization methodologies are well described in “Our optimization methodology” section.

We exploited an approximate HE scheme HEAAN [3, 4] with a publicly available library [5] for the implementation of our modified semi-parallel GWAS algorithm based on HE. The HE algorithm takes about 40 minutes for 245 samples each containing a binary phenotype, 3 covariates, and 14,841 SNPs on Linux with a 2.10GHz processor using 8 threads (4 cores).

### Related works

Some works on HE-based genome analyses has been studied over the past years: Lauter et al. [6] studied application of HE on basic genomic algorithms such as the Pearson Goodness-of-Fit test, and Wang et al. [7] performed an exact logistic regression on small datasets based on HE. More recently, some solutions [8–11] submitted to task 3 of iDASH 2017 [12] competition dealt with training the logistic regression model of genomic data based on HE. Some works [13, 14] studied the privacy-preserving GWAS based on HE, but they performed rather simple *χ*^2^ test on quite small numbers of SNPs, which is quite different from iDASH 2018 task; logistic regression on large numbers of SNPs.

There are some other alternative tools to deal with privacy issues in real-world applications. One of them is a cryptographic tool called secure Multi-Party Computation (MPC). In MPC, computations are done online by multiple parties without revealing any information of the result to each party. There have been several remarkable works on privacy-preserving genome analysis based on MPC. In 2017, Jagadeesh et al. [15] proposed privacy-preserving solutions for several genomic diagnose methods based on MPC with practical implementation over real patient data. Recently, Cho, Wu and Berger [16] successfully constructed a practical MPC-based protocol for privacy-preserving GWAS computation over large-scale genomic data. There have been several previous works [14, 17–19] on privacy-preserving GWAS computation based on MPC; however, they had some limitations to be applied in practice since they either require infeasible computational cost or greatly streamlined the task.

Meanwhile, in 2016, Chen et al. [20] proposed a hardware-based solution for privacy-preserving genome analysis with Software Guard Extensions (SGX) [21], the security-related instruction built in Intel CPU which allows secure computations in a private region which cannot be accessed without the private key. They implemented a SGX-based framework for secure transmission disequilibrium test on Kawasaki disease patients with high scalability on the number of SNPs.

## Methods

### Approximate homomorphic encryption scheme HEAAN

For privacy-preserving GWAS computation, we applied an HE scheme called HEAAN proposed by Cheon et al. [3, 4], which supports *approximate computation* of real numbers in encrypted state. Efficiency of HEAAN in the real world has been proved by showing its application in various fields including machine learning [8, 22, 23] and cyber physical system [24]. The winning solution of iDASH competition in 2017 also applied HEAAN as an HE scheme for privacy-preserving logistic regression on genomic data.

At high-level, for a HEAAN ciphertext *ct* of some message polynomial ${\mathfrak {m}}$, the decryption process with a secret key *sk* is done as

$${\texttt{Dec}}_{{\mathsf{sk}}}({\mathsf{ct}}) = {\mathfrak{m}} + e \approx {\mathfrak{m}}$$

where *e* is a small error attached to the message polynomial ${\mathfrak {m}}$. Furthermore, for ciphertexts *ct*_1_ and *ct*_2_ of message polynomials ${\mathfrak {m}}_{1}$ and ${\mathfrak {m}}_{2},$ the homomorphic evaluation algorithms C.Add and C.Mult satisfy

$$\begin{array}{@{}rcl@{}} {\texttt{Dec}}_{{\mathsf{sk}}}\left({\texttt{C.Add}}({\mathsf{ct}}_{1}, {\mathsf{ct}}_{2})\right) &\approx& {\mathfrak{m}}_{1} + {\mathfrak{m}}_{2},\\ {\texttt{Dec}}_{{\mathsf{sk}}}({\texttt{C.Mult}}_{{\mathsf{evk}}}\left({\mathsf{ct}}_{1}, {\mathsf{ct}}_{2})\right) &\approx& {\mathfrak{m}}_{1} \cdot {\mathfrak{m}}_{2}, \end{array} $$

i.e., addition and multiplication can be *internally* done even in encrypted state.

For formal definitions, let *L* be a level parameter, and *q*_*ℓ*_:=2^*ℓ*^ for 1≤*ℓ*≤*L*. Let $R := {\mathbb {Z}}[X]/\left (X^{N}+1\right)$ for a power-of-two *N* and *R*_*q*_ be a modulo-*q* quotient ring of *R*, i.e., *R*_*q*_:=*R*/*qR*. The distribution *χ*_key_:=*HW*(*h*) over *R*_*q*_ outputs a polynomial of {−1,0,1}-coefficient having h number of non-zero coefficients, and *χ*_enc_ and *χ*_err_ denote the discrete Gaussian distribution with some prefixed standard deviation. We denote the rounding function by ⌊·⌉, which outputs the nearest integer of a real-number input. For $a=\sum _{i=0}^{N-1}a_{i}X^{i}\in {\mathbb {R}}[X]/\left (X^{N}+1\right)$, then $\lfloor a \rceil := \sum _{i=0}^{N-1}\lfloor a_{i} \rceil X^{i} \in R$. Finally, [·]_*q*_ denotes a component-wise modulo *q* operation on each element of *R*_*q*_. Note that those parameters *N*, *L* and h satisfying a certain security level can be determined by Albrecht’s security estimator [25, 26]. The scheme description of HEAAN is as following:

- $\underline {{\texttt {KeyGen}}({\mathsf {params}})}.$
  - Sample *s*←*χ*_key_. Set the secret key as *sk*←(1,*s*).
  - Sample $a\leftarrow U\left (R_{q_{L}}\right)$ and *e*←*χ*_err_. Set the public key as ${\mathsf {pk}}\leftarrow (b,a)\in R_{q_{L}}^{2}$ where $b\leftarrow [-a\cdot s+e]_{q_{L}}$.
  - Sample $a'\leftarrow U\left (R_{q_{L}^{2}}\right)$ and *e*^′^←*χ*_err_. Set the evaluation key as ${\mathsf {evk}}\leftarrow \left (b',a'\right)\in R_{q_{L}^{2}}^{2}$ where $b'\leftarrow \left [-a's+e'+q_{L}\cdot s^{2}\right ]_{q_{L}^{2}}$.
- $\underline {{\texttt {Enc}}_{{\mathsf {pk}}}({\mathfrak {m}})}$. For a message ${\mathfrak {m}}\in R$, sample *v*←*χ*_enc_ and *e*_0_,*e*_1_←*χ*_err_. Output the ciphertext ${\mathsf {ct}} = \left [v\cdot {\mathsf {pk}}+({\mathfrak {m}}+e_{0},e_{1})\right ]_{q_{L}}$.
- $\underline {{\texttt {Dec}}_{{\mathsf {sk}}}({\mathsf {ct}})}$. For a ciphertext ${\mathsf {ct}}= (c_{0},c_{1})\in R_{q_{\ell }}^{2}$, output a message ${\mathfrak {m}}'=[c_{0} + c_{1}\cdot s]_{q_{\ell }}.$
- $\underline {{\texttt {C.Add}}\left ({\mathsf {ct}},{\mathsf {ct}}'\right)}$. For ${\mathsf {ct}},{\mathsf {ct}}'\in R_{q_{\ell }}^{2}$, output ${\mathsf {ct}}_{{\texttt {add}}}\leftarrow \left [{\mathsf {ct}}+{\mathsf {ct}}'\right ]_{q_{\ell }}$.
- $\underline {{\texttt {C.Sub}}\left ({\mathsf {ct}},{\mathsf {ct}}'\right)}$. For ${\mathsf {ct}},{\mathsf {ct}}'\in R_{q_{\ell }}^{2}$, output ${\mathsf {ct}}_{{\texttt {sub}}}\leftarrow \left [{\mathsf {ct}}-{\mathsf {ct}}'\right ]_{q_{\ell }}$.
- $\underline {{\texttt {C.Mult}}_{{\mathsf {evk}}}\left ({\mathsf {ct}},{\mathsf {ct}}'\right)}$. For ${\mathsf {ct}}=(c_{0},c_{1}),{\mathsf {ct}}'=\left (c_{0}',c_{1}'\right)\in {\mathcal R}_{q_{\ell }}^{2}$, let (*d*_0_,*d*_1_,*d*_2_)=(*c*_0_*c*0′,*c*_0_*c*1′+*c*_1_*c*0′,*c*_1_*c*1′). Output ${\mathsf {ct}}_{{\texttt {mult}}}\leftarrow \left [\left (d_{0}, d_{1}\right)+\left \lfloor {q_{L}^{-1}\cdot d_{2}\cdot {\mathsf {evk}} }\right \rceil \right ]_{q_{\ell }}$.

For more details of the scheme including the correctness and security analysis, we refer the readers to [3].

The above-mentioned scheme deals with message polynomial ${\mathfrak {m}}$ in some integer polynomial ring ${\mathbb {R}}.$ To encrypt real (or complex) value, HEAAN use a (field) isomorphism $\tau : {\mathbb {R}}[X]/\left (X^{N}+1\right) \rightarrow {\mathbb {C}}^{N/2}$ called canonical embedding. A plaintext vector $\vec m = (m_{0},..., m_{N/2 - 1})$ is first transformed into $\tau ^{-1}\left (\vec m\right) \in {\mathbb {R}}[X] / \left (X^{N} + 1\right)$, and then rounded off to an integer-coefficient polynomial. However, the naive rounding-off $\left \lfloor {\tau ^{-1}\left (\vec m\right)}\right \rceil $ can derive quite large relative error on the plaintext. To control the error, we round it off after scaling up by *p* bits for some integer *p*, i.e., $\left \lfloor {2^{p} \cdot \tau ^{-1}\left (\vec m\right)}\right \rceil $, so that the relative error is reduced. Clearly, a decoding algorithm for ${\mathfrak {m}}$ would be $2^{-p} \cdot \tau ({\mathfrak {m}})$:

- $\underline {{\texttt {Ecd}}\left (\vec m;p\right)}$. For $\vec m=(m_{0},...,m_{N/2-1})$ in ${\mathbb {C}}^{N/2}$ and a precision bit *p*>0, output a polynomial ${\mathfrak {m}} \leftarrow \left \lfloor {2^{p}\cdot \tau ^{-1}\left (\vec m\right)}\right \rceil \in R$ where the rounding ⌊·⌉ is done coefficient-wisely.
- $\underline {{\texttt {Dcd}}({\mathfrak {m}};p)}$. For ${\mathfrak {m}} \in R$, output a plaintext vector $\vec m' = 2^{-p}\cdot \tau ({\mathfrak {m}}) \in {\mathbb {C}}^{N/2}$.

To sum up, to encrypt a plaintext vector of real (complex) numbers $\vec m$, we first encode $\vec m$ into ${\mathfrak {m}} \leftarrow {\texttt {Ecd}}\left (\vec m ;p\right)$ with a certain precision bit *p*, and then generate a ciphertext ${\mathsf {ct}} \leftarrow {\texttt {Enc}}_{{\mathsf {pk}}}({\mathfrak {m}})$ with the public key *pk*.

Now consider *ct*_1_ and *ct*_2_ be ciphertexts of $\vec m_{1}$ and $\vec m_{2}$ in ${\mathbb {C}}^{N/2}.$ Since our encoding method scales each plaintext vector up by 2^*p*^, the plaintext vector of a ciphertext *ct*^′^←C.Mult_*evk*_(*ct*_1_,*ct*_2_) is (approximately) $2^{p} \cdot \vec m_{1} \odot \vec m_{2},$ not $\vec m_{1} \odot \vec m_{2}$, which will result in exponential growth of plaintexts. To deal with this problem, we adjust the scaling factor by the following procedure so-called rescaling:

- $\underline {{\texttt {RS}}_{\ell \rightarrow \ell '}({\mathsf {ct}})}$. For a ciphertext ${\mathsf {ct}}\in R_{q_{\ell }}^{2}$, output ${\mathsf {ct}}'\leftarrow \left [\lfloor {\left (q_{\ell '}/q_{\ell }\right)\cdot {\mathsf {ct}}}\rceil \right ]_{q_{\ell '}}$.

After the rescaling procedure *ct*_mult_←RS(*ct*^′^), the plaintext vector of the output *ct*_mult_ is (approximately) $\vec m_{1} \odot \vec m_{2}$, and the ciphertext modulus *q*_*L*_ is reduced by 2^*p*^. As a result, the level parameter *L* should be carefully chosen according to the multiplicative depth of a target computation. In order to present our algorithm in a simple form, we will not describe these rescaling procedures for the rest of the paper, but we remark that in the actual use of *HEAAN*, there should be delicate consideration on scaling of message.

To deal with a plaintext vector of the form $\vec m \in {\mathbb {C}}^{k}$ having length *K*≤*N*/2 for some power-of-two divisor *K* of *N*/2,*HEAAN* encrypts $\vec m$ into a ciphertext of an *N*/2-dimensional vector $\left (\vec m || \cdots || \vec m\right) \in {\mathbb {C}}^{N/2},$ where $\left (\vec v || \vec w\right)$ denotes the concatenation of two vectors $\vec v$ and $\vec w$. This implies that a ciphertext of $\vec m \in {\mathbb {C}}^{k}$ can be understood as a ciphertext of $\left (\vec m || \cdots || \vec m \right) \in {\mathbb {C}}^{K'}$ for powers-of-two *K* and *K*^′^ satisfying *K*≤*K*^′^≤*N*/2.

Finally, the HEAAN scheme provides the rotation operation on plaintext slots, i.e., it enables us to securely obtain an encryption of the shifted plaintext vector $\left (m_{r},\dots,m_{N/2-1},m_{0},\dots,m_{r-1}\right)$ from an encryption of $\left (m_{0},\dots,m_{N/2-1}\right)$. It is necessary to generate an additional public information *rk*, called the rotation key. We denote the rotation operation as follows.

- $\underline {{\texttt {Rot}}_{{\mathsf {rk}}}({\mathsf {ct}}; r)}$. For the rotation key *rk*, output a ciphertext *ct*^′^ encrypting the (left) rotated plaintext vector of *ct* by *r*(>0) positions as above example. If *r*<0, it denotes the right rotation by (−*r*) positions.

We omit a subscript of each algorithm of HEAAN for convenience if it is obvious.

### Matrix packing method and rotate function

In this subsection, we describe an encoding method to encrypt a matrix structure in a ciphertext which was also introduced in [8]. Consider an *n*×*m* matrix *Z*

$$Z=\left[\begin{array}{ccc} z_{0,0} & \cdots & z_{0,m-1} \\ \vdots & \ddots & \vdots \\ z_{n-1,0} & \cdots & z_{n-1,m-1} \end{array}\right]. $$

We first pad zeros to set the number of rows and columns to be powers-of-two, say $\overline n$ and $\overline m,$ and assume that $\log \overline n +\log \overline m\le \log (N/2)$. Then we pack the whole matrix in a single ciphertext *ct*_*Z*_ in a column-by-column manner. As described above, the algorithm Rot(*ct*_*Z*_;*r*) can shift the encrypted vector by *r* positions. In particular, we can perform row and column rotations of an encrypted matrix with this operation. When $r=\overline n\cdot j$, and the result will be the (left) column rotation of the encrypted matrix *Z* by *j* columns.

For the row rotation of an encrypted matrix, we use so-called *masking approach*. Consider *n*×*m* matrices *M*_*i*_ and $\overline {M_{i}}$, where the first *n*−*i* rows of *M*_*i*_ (resp. $\overline {M_{i}}$) are filled with 1 (resp. 0) and the last *i* rows of *M*_*i*_ (resp. $\overline {M_{i}}$) are filled with 0 (resp. 1). Let *msk*_*i*_ and $\overline {{\mathsf {msk}}_{i}}$ be the ciphertext of *M*_*i*_ and $\overline {M_{i}}$, respectively.

$$M_{i}=\left[\begin{array}{ccc} 1 & \cdots & 1 \\ 1 & \cdots & 1 \\ \vdots & \ddots & \vdots \\ 0 & \cdots & 0 \end{array}\right] ~,~~ \overline{M_{i}} = \left[\begin{array}{ccc} 0 & \cdots & 0 \\ 0 & \cdots & 0 \\ \vdots & \ddots & \vdots \\ 1 & \cdots & 1 \end{array}\right] ~~ $$

To rotate *Z* by *i* rows in encrypted state, we first compute *ct*_1_←Rot(*ct*_*Z*_,*i*) and *ct*_2_←Rot(*ct*_*Z*_,*i*−*n*). Then, we mask them as *ct*_1_←C.Mult(*ct*_1_,*msk*_*i*_) and ${\mathsf {ct}}_{2} \leftarrow {\texttt {C.Mult}}\left ({\mathsf {ct}}_{2}, \overline {{\mathsf {msk}}_{i}}\right)$. As a result, the output of C.Add(*ct*_1_,*ct*_2_) is a ciphertext of upper row rotation of *Z* by *i* rows.

Those row and column rotations of an encrypted matrix are denoted as follows:

- $\underline {{\texttt {C.ColumnRot}}\left ({\mathsf {ct}}_{Z},j\right)}$. For a ciphertext *ct*_*Z*_ of a matrix *Z* and an integer *j*, output a ciphertext *ct* of left column rotation of *Z* by *j* columns.
- $\underline {{\texttt {C.RowRot}}\left ({\mathsf {ct}}_{Z},i\right)}$. For a ciphertext *ct*_*Z*_ of a matrix *Z* and an integer *j*, output a ciphertext *ct* of upper row rotation of *Z* by *i* rows.

### Semi-parallel GWAS algorithm

A naive application of GWAS analysis can be done by running a logistic regression for each SNP, which resulting in high computational cost since the number of SNPs can be usually hundred thousands or more. To overcome this problem, Sikorska *et al.*[27] proposed a semi-parallel GWAS algorithm which reduces the required computation time from 6 hours to 10-15 minutes using projections.

Let *n* be the number of samples each of which consists of *m* (binary) SNP data and *k*^′^ covariate data. Then the whole SNP data and covariate data can be organized as an *n*×*m* matrix *S* and an *n*×*k*^′^ matrix *X*_0_, respectively. For *k*:=*k*^′^+1, we define a matrix *X* as the concatenation of a vector whose components are 1 and *X*_0_, denoted by $X = \left (\vec {1}|| X_{0}\right)$. Let $\vec y$ be a target binary phenotype vector of length *n*. With these inputs, the semi-parallel GWAS algorithm outputs the *m*-dimensional vector $\overrightarrow {\textsf {pval}}$ which indicates the *p*-value of each SNP with respect to the target phenotype. The detail of the algorithm is described in Algorithm 1.


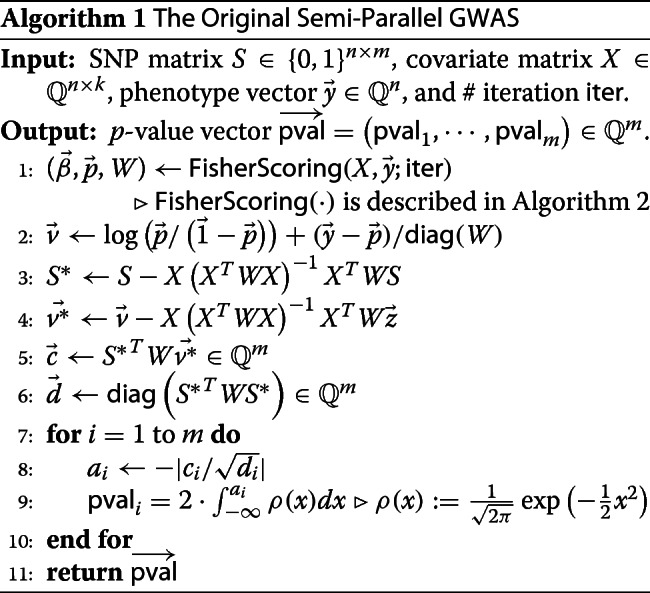


The semi-parallel GWAS algorithm involves logistic regression on $\left (X,{\vec y}\right)$ in the first step, and Fisher Scoring [28] described in Algorithm 2 is one of the most highly efficient algorithm for logistic regression.


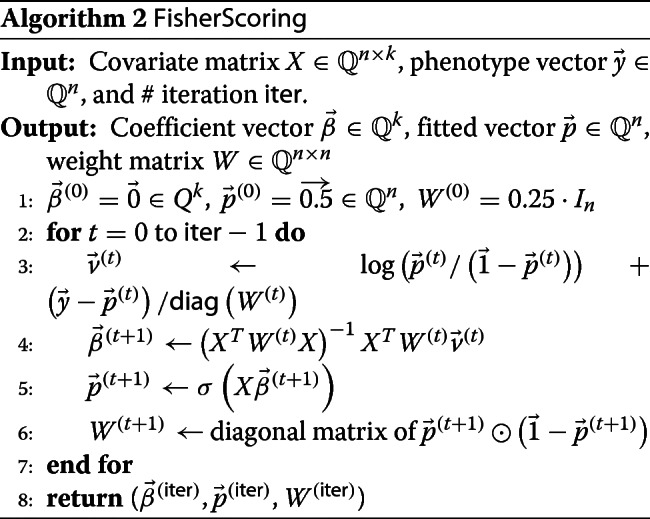


In both algorithms, *σ*(*x*):=1/(1+ exp(−*x*)) is called sigmoid function. For both algorithms, if the input of functions such as logarithm (log), division (/) and sigmoid (*σ*) is a vector, then it means to apply the function component-wisely resulting in the output vector of the same length. The notation *T* in the superscript denotes the matrix transpose. The operation ⊙ denotes the Hadamard (component-wise) multiplication of two vectors, and the notation diag(·) with an input of a square matrix means the diagonal vector of the input.

### Our optimization methodology

The aim of this study is to construct an HE algorithm for privacy-preserving semi-parallel GWAS computation. Since non-polynomial operations such as matrix inverse or real number inversion is a challenging stuff in HE, we need to modify the original semi-parallel GWAS algorithm into HE-friendly form for efficiency. Moreover, the super-large data size of GWAS requires too much computational cost in encrypted state, and this issue should be resolved. In this regard, we introduce our optimization methodology to the algorithm.

#### Modification of fisher scoring

The main obstacle of Fisher Scoring (Algorithm 2) is a matrix inversion for $U = X^{T}WX \in {\mathbb {Q}}^{k\times k}.$ To overcome this problem, we exploit the adjoint matrix adj(*U*) and the determinant det(*U*) of *U*. For (*i*,*j*)-minor $M_{i,j}\in {\mathbb {Q}}$ of *U*, adj(*U*) and det(*U*) are obtained from basic linear algebra:

1 $$\begin{array}{@{}rcl@{}} {\textsf{adj}}(U) &=& \left[(-1)^{i+j}\cdot M_{i,j} \right] \in {\mathbb{Q}}^{k\times k}, \end{array} $$

2 $$\begin{array}{@{}rcl@{}} {\textsf{det}}(U) &=&\sum\limits_{i=0}^{k-1}u_{i,0}\cdot (-1)^{i} \cdot M_{i,0}. \end{array} $$

We express the inverse matrix *U*^−1^ as $U^{-1} = \frac {1}{{\textsf {det}}(U)}\cdot {\textsf {adj}}(U).$ To be precise, observe that

$$\begin{array}{@{}rcl@{}} {\vec v}^{(t)} &=& \log\left(\frac{{\vec p}^{(t)}}{\vec 1-{\vec p}^{(t)}} \right) + \frac{{\vec y} - {\vec p}^{(t)}}{{\textsf{diag}}\left(W^{(t)}\right)} \\ &=& X{\vec \beta}^{(t)} + \frac{{\vec y} - {\vec p}^{(t)}}{{\textsf{diag}}\left(W^{(t)}\right)}, \end{array} $$

from which we obtain an iterative updating equation on ${\vec \beta }^{(t)}$ as follows:

$$\begin{array}{@{}rcl@{}} {\vec \beta}^{(t+1)} &=& U^{-1}X^{T}W^{(t)} \left(X{\vec \beta}^{(t)} + \frac{{\vec y} - {\vec p}^{(t)} }{{\textsf{diag}}\left(W^{(t)}\right) }\right)\\ &=& {\vec \beta}^{(t)} + U^{-1}X^{T}\left({\vec y} - {\vec p}^{(t)} \right)\\ &=&{\vec \beta}^{(t)} + \frac{1}{{\textsf{det}}(U)}\cdot{\textsf{adj}}(U)X^{T}\left({\vec y} - {\vec p}^{(t)} \right). \end{array} $$

Here one needs to compute the inverse of det(*U*), but this non-polynomial operation is rather expensive in HE. The key observation on this equation is that the second term $U^{-1}X^{T}\left ({\vec y} - {\vec p}^{(t)}\right)$ essentially converges to 0 as *t*→*∞* since ${\vec \beta }^{(t)}$ converges to some point. From this, we may expect that the convergence would be still valid even when we neglect the term det(*U*)^−1^ and substitute it by some appropriate constant. Namely, we can modify the equation as

$${\vec \beta}^{(t+1)} = {\vec \beta}^{(t)} + \alpha\cdot{\textsf{adj}}(U)X^{T}\left({\vec y} - {\vec p}^{(t)}\right). $$

for some constant *α*>0. In practice, this approximate version of the Fisher scoring algorithm works quite well with slightly slower convergence rate.

#### Computing diag(*S*^∗^^*T*^*WS*^∗^) without *S*^∗^

The main observation of this subsection is that computation of *n*×*m* matrix *S*^∗^ in Algorithm 1 is superfluous for obtaining *p*-values. Rather than computing *S*^∗^, we directly compute det(*U*)·diag(*S*^∗^^*T*^*WS*^∗^), which can be obtained without computing (matrix) inversion. To be precise, using the fact that *S*^∗^=*S*−*XU*^−1^*V* for *V*:=*X*^*T*^*WS*, we get

$$\begin{array}{@{}rcl@{}} {S^{*}}^{T}WS^{*} &=& \left(S - XU^{-1}V\right)^{T} W \left(S - XU^{-1}V\right)\\ &=& S^{T}WS - V^{T}U^{-1}V. \end{array} $$

Based on this observation, we compute det(*U*)·diag(*S*^∗^^*T*^*WS*^∗^) by following:

1. Compute *U*=*X*^*T*^*WX* and *V*=*X*^*T*^*WS*.
2. Compute adj(*U*) and det(*U*).
3. Compute det(*U*)·diag(*S*^*T*^*WS*)−diag(*V*^*T*^adj(*U*)*V*).

#### Approximate computation of ${S^{*}}^{T}W{\vec {v^{*}}}$

We also take the main observation of the previous subsection so that we do not compute *S*^∗^. From the definition of *S*^∗^ and ${\vec {v^{*}}}$, it holds that ${S^{*}}^{T}W{\vec {v^{*}}} = S^{T}W{\vec {v^{*}}}$. Then we have the following equations:

$$\begin{array}{@{}rcl@{}} S^{T}W{\vec{v^{*}}} &=& S^{T}W\left(I - XU^{-1}X^{T}W\right){\vec v}\\ &=&S^{T}W\left(I - XU^{-1}X^{T}W\right)\frac{{\vec y}-{\vec p}}{{\textsf{diag}}(W)}\\ &=& S^{T}\left({\vec y}-{\vec p}\right) -S^{T}WXU^{-1}X^{T}\left({\vec y}-{\vec p}\right)\\ &\simeq& S^{T}\left({\vec y}-{\vec p}\right) \end{array} $$

where the last approximation is valid since the term $X^{T}\left ({\vec y}-{\vec p}\right)$ is sufficiently close to the zero vector, which is resulted from the Fisher Scoring. For example, each component of $X^{T}\left ({\vec y}-{\vec p}\right)$ was approximately 10^−30^ in our experiment with 4 iterations in Fisher scoring. Refer to “Dataset description” section for the specific description of datasets. Therefore, we compute ${\textsf {det}}(U) \cdot S^{T}\left ({\vec y}-{\vec p}\right)$ which is a reliable approximation of ${\textsf {det}}(U)\cdot {S^{*}}^{T}W{\vec {v^{*}}},$ in much less computational costs.

#### Our modified semi-parallel GWAS algorithm

To sum up all our algorithmic optimization techniques described in above, we have Algorithm 3 and 4, which are modified Fisher Scoring and semi-parallel GWAS algorithms, respectively.


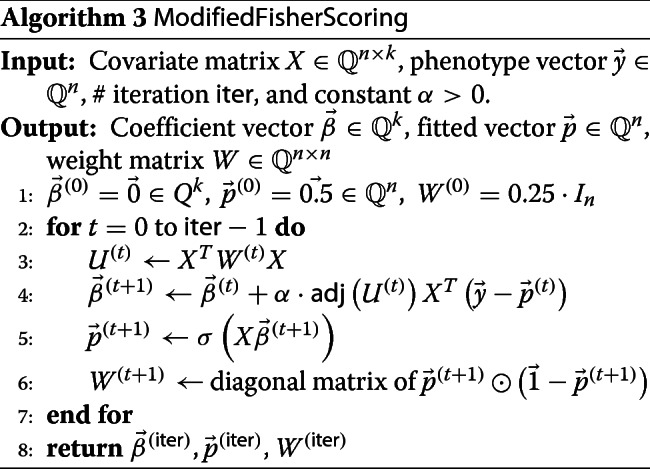


In Algorithm 3, the constant *α* takes a similar role to the learning rate in gradient descent algorithm [29], which can be adjusted if necessary.


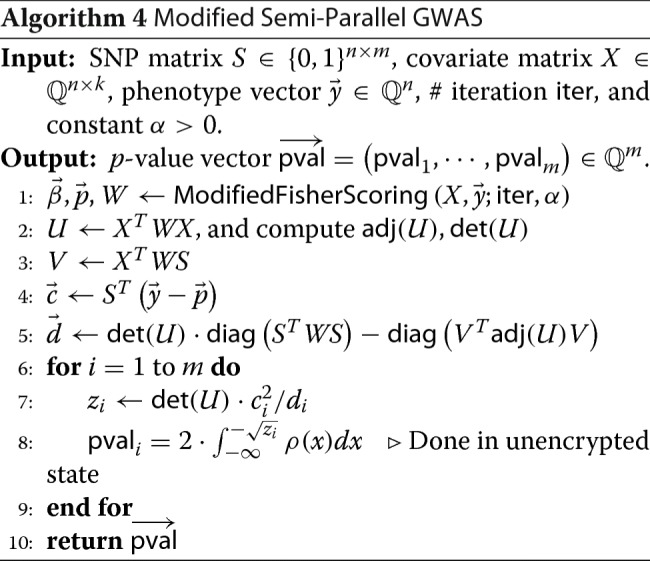


We remark that step 8 of Algorithm 4, a conversion procedure from the squared *z*-score *z*_*i*_ to the *p*-value pval_*i*_, is done in unencrypted state. Namely, we decrypt the ciphertext of *z*_*i*_ for 1≤*i*≤*m* after step 7 so that *z*_*i*_’s are publicized. We stress that the squared *z*-score has exactly *the same information* as the *p*-value, so publishing squared *z*-scores does not leak any additional information more than publishing *p*-values.

### Homomorphic evaluation of the modified semi-parallel GWAS algorithm

Upon HE-friendly algorithms discussed in the previous section, there still remain computational issues regarding more fundamental operations. Recall that HEAAN basically supports component-wise addition and multiplication along with data slot rotations. However, we encrypt the data matrix by column-by-column manner, and our algorithms include complex operations such as matrix multiplication, evaluation of the adjoint matrix, a sigmoid function, and so on. In this regard, we specify how we can deal with such operations efficiently, reducing the number of multiplications or the total depth of multiplications required which are the main bottleneck of HE.

Note that this section consists of rather technical contents related to HE, since it includes HE algorithms of all building blocks for Algorithm 3 and Algorithm 4. One can simply embrace the fact that every operation required in Algorithm 3 and Algorithm 4 can be efficiently done based on HE, if not really interested in the details.

Hereafter, [*a*]_*k*_ with an integer *a* denotes a residue number in [0,*k*−1] modulo *k*. An *n*-dimensional vector $\vec a = (a_{1}, \cdots, a_{n})$ is simply denoted by (*a*_*i*_)_1≤*i*≤*n*_, and an *n*×*m* matrix *A* having (*i*,*j*)-entry *a*_*i*,*j*_ is denoted by [*a*_*i*,*j*_]_1≤*i*≤*n*,1≤*j*≤*m*_. For both cases, if the size is obvious from context, we simply write a vector by (*a*_*i*_), and a matrix by [*a*_*i*,*j*_]. Every vector and matrix in this section is assumed to be of size power-of-two, which is in line with our packing method introduced in “Matrix packing method and rotate function” section.

#### Adjoint matrix and determinant

In step 4 of Algorithm 3 and step 2 of Algorithm 4, we need to compute the adjoint matrix and the determinant of the matrix *U*=[*u*_*i*,*j*_]_*i*,*j*_=*X*^*T*^*WX*.

We recall Eqs. 1 and 2 for adjoint matrix and determinant. Given an encryption of *U*, denoted by *C*_*U*_, we generate (*k*−1)^2^ ciphertexts *C*_*i*,*j*_ for 1≤*i*,*j*≤*k*−1 from *C*_*U*_, whose plaintext is an *i*-row (upper) rotation and *j*-column (left) rotation of *U*. We first consider the 0-th plaintext slot, i.e., the (0,0)-position of the plaintext matrix, of the ciphertexts. Since every *u*_*a*,*b*_ for 1≤*a*,*b*≤*k*−1 is a (0,0)-entry of plaintext matrix for one and only one ciphertext *C*_*a*,*b*_, we can compute a ciphertext whose (0,0)-entry of the plaintext matrix is *M*_0,0_ from *C*_*a*,*b*_’s, by homomorphically evaluating the polynomial *f* which outputs *M*_0,0_ with input *u*_*a*,*b*_’s.

Now observe that *M*_*a*,*b*_ and $\phantom {\dot {i}\!}M_{a',b'}$ have a formula of the same form where the subscript indices of *u*_*i*,*j*_ are shifted by (*a*^′^−*a*,*b*^′^−*b*) modulo *k*. Thanks to this index-shifting property, the homomorphic evaluation of the polynomial *f* with input *C*_*a*,*b*_’s essentially outputs a ciphertext of which the plaintext matrix is [*M*_*i*,*j*_].

After computing the ciphertext of [*M*_*i*,*j*_] as above, we can obtain the ciphertext *C*_adj_ of adj(*U*) by multiplying a ciphertext *C*_sgn_ of [(−1)^*i*+*j*^]. Finally, the ciphertext *C*_det_ of determinant det(*U*) is easily obtained from the homomorphic multiplication of *C*_*U*_ and *C*_adj_, followed by log*k* rotations and summations.

In case of *k*=4, for example, the polynomial *f* is defined as *f*([*u*_*i*,*j*_]_1≤*i*,*j*≤3_)=*u*_1,1_*u*_2,2_*u*_3,3_−*u*_1,1_*u*_2,3_*u*_3,2_−*u*_2,1_*u*_1,2_*u*_3,3_+*u*_2,1_*u*_1,3_*u*_3,2_+*u*_3,1_*u*_1,2_*u*_2,3_−*u*_3,1_*u*_1,3_*u*_2,2_. Then, the homomorphic evaluation to obtain *C*_adj_ is done as Algorithm 5.


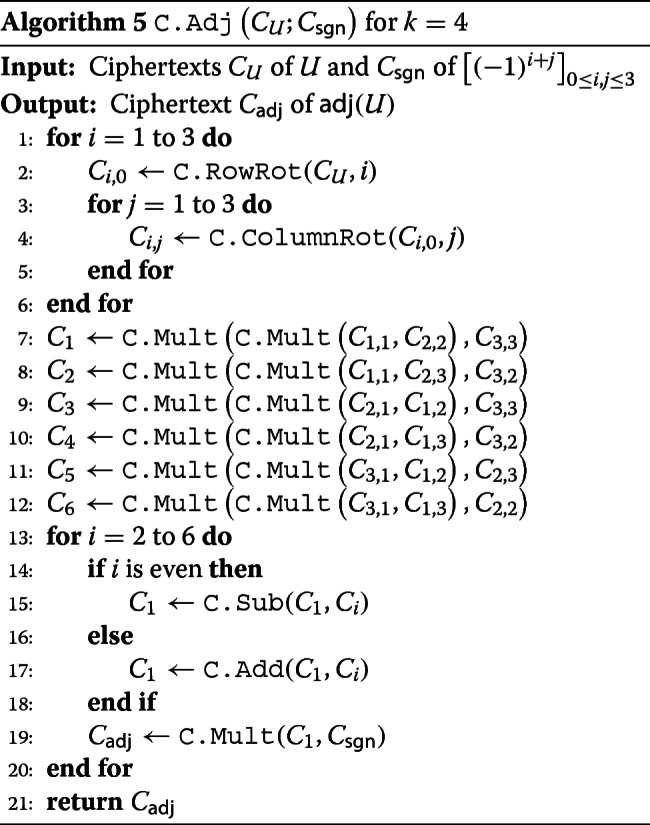


#### Matrix multiplications

Let *A*=[*a*_*i*,*j*_] be an *n*×*k* matrix with *n*≥*k* and *B*=[*b*_*i*,*j*_] be an *n*×*m* matrix. We use an Algorithm 6 computing a ciphertext $\phantom {\dot {i}\!}C_{A^{T}B}$ of *A*^*T*^*B* from ciphertexts *C*_*A*_ and *C*_*B*_ of *A* and *B*, which is inspired from the hybrid method by Juvekar et al. [30].

As the first step, we compute *k* ciphertexts of

$${\textsf{diag}}_{t}(A) = \left(a_{i, [i-t]_{k}}\right)_{0 \le i \le n-1}$$

for 0≤*t*≤*k*−1. For this, we use ciphertexts *dmsk*_*t*_ of *n*×*k* masking matrix, of which the (*i*,*j*)-entry is $\delta _{[i+t]_{k}, j}.$ Here *δ*_*i*,*j*_ denotes the Kronecker Delta. By summing column rotations of *A*⊙*dmsk*_*t*_, we get ciphertexts of *n*×*m* matrices *Expdiag*_*t*_(*A*) having *m* identical columns diag_*t*_(*A*). Then, we compute the following matrix *M*:

$$\begin{array}{@{}rcl@{}} M &=& \sum\limits_{t=0}^{k-1} \rho_{t}\left({\sf{Expdiag}}_{t}(A) \odot B\right) \\ &=& \sum\limits_{t=0}^{k-1} \rho_{t} \left(\left[ a_{i, [i-t]_{k}} \cdot b_{i,j} \right]\right) \\ &=& \sum\limits_{t=0}^{k-1} \left[ a_{[i+t]_{n},[i]_{k}} \cdot b_{[i+t]_{n},j} \right] \in {\mathbb{Q}}^{n \times m}, \end{array} $$

where *ρ*_*t*_ is an (upward) *t*-rotation of matrix by row. Thus, by properly summing rows of *M*, we obtain $A^{T} B = \sum _{t=0}^{n-1} [a_{t,i} \cdot b_{t,j}] \in {\mathbb {Q}}^{k \times m}.$ The detail of this algorithm is described in Algorithm 6.


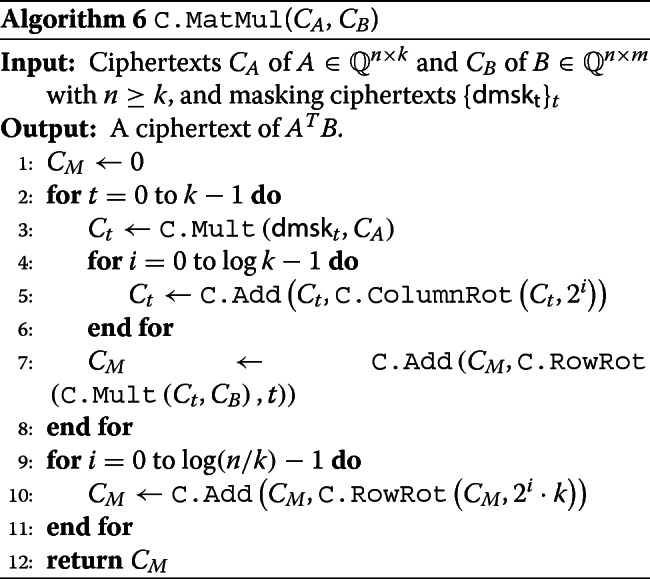


Indeed, our GWAS algorithm contains several matrix multiplications of the form *A*^*T*^*DB* for the diagonal matrix *D*. For this, we first compute *B*^′^=*DB* by *Expdiag*_0_(*D*)⊙*B*, and then obtain *A*^*T*^*DB* by computing *A*^*T*^*B*^′^ with the above method. Note that this requires only one additional Hadamard multiplication.

#### Matrix-vector multiplications

By understanding a vector by an one column matrix, we can perform all matrix-vector multiplications in our algorithms, except $X{\vec \beta }$ that appears in step 5 of Algorithm 3.

In fact, we can also compute $X{\vec \beta }$ by changing Algorithm 6 a little bit. Recall that *X*=[*x*_*i*,*j*_] is an *n*·*k* matrix and ${\vec \beta }=(\beta _{i})$ is a *k*-length vector, and it holds that *n*≥*k*. We now again compute *k* ciphertexts of diag_*t*_(*X*), and then compute

$$\begin{aligned} &\sum\limits_{t=0}^{k-1} {\textsf{diag}}_{t}(X) \odot \rho_{-t}\left(\left({\vec \beta} || \cdots ||{\vec \beta}\right) \right) \\ &= \sum\limits_{t=0}^{k-1} \left(x_{i, [i-t]_{k}}\right) \odot \left(\beta_{[i-t]_{k}}\right) \\ &= \sum\limits_{t=0}^{k-1} \left(x_{i, [i-t]_{k}} \cdot \beta_{[i-t]_{k}}\right) = X {\vec \beta}, \end{aligned} $$

whose algorithm is described by Algorithm 7.


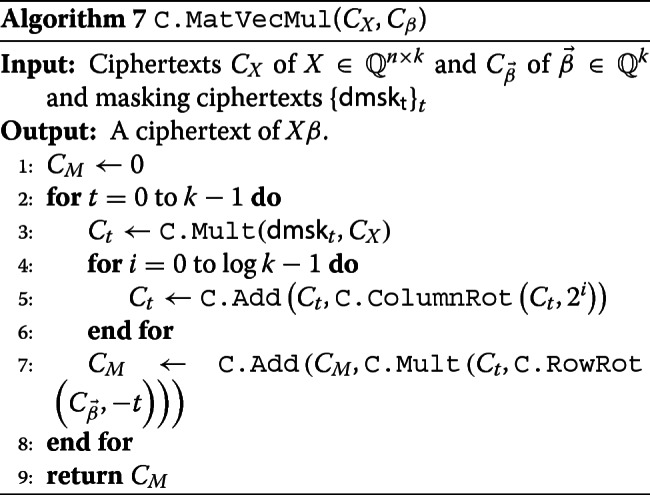


We remark that there is another simple method for matrix-vector multiplication that we use for $\vec c = S^{T} \left (\vec y - \vec p\right)$. For simplicity, let $ \vec x = (x_{i}) := \vec y - \vec p.$ Then by rotating and summing all rows of $S\odot \left [\vec x || \cdots || \vec x\right ] = \left [s_{i,j}\cdot x_{i}\right ],$ we obtain a matrix having the same size with *S* and consisting of identical *rows*$ \vec c = S^{T} \vec x.$ This requires only one Hadamard multiplication and log*n* rotations. However, strictly speaking, this resulting ciphertext is not a ciphertext of $S^{T} \vec x,$ since it encrypts a matrix having $\vec c$ row-wisely, not column-wisely. Thus we can only use this simple method only for $S^{T} \vec x,$ where this row-wise packing does not matter after then. The detail of this algorithm is described in Algorithm 8.


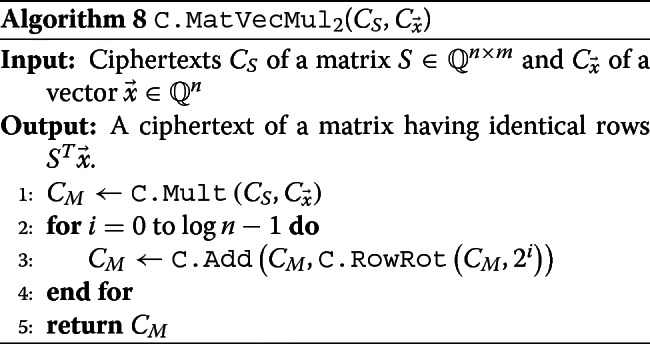


#### Fast diag(*A*^*T*^*BA*) computations

To obtain diag(*A*^*T*^*BA*), one can perform matrix multiplication followed by diagonal extraction, but it is obviously not optimal since this computes unnecessary entries of *A*^*T*^*BC* other than diagonal entries. Thus we use another method that only compute the diagonal entries.

Let *A*=[*a*_*i*,*j*_] be an *n*×*m* matrix. As an incremental step, we first consider diag(*A*^*T*^*DC*) where *D*=[*d*_*i*,*j*_] is an *n*×*n* diagonal matrix and *C*=[*c*_*i*,*j*_] is an *n*×*m* matrix. Then it holds that ${\textsf {diag}}\left (A^{T}DC\right)_{j} = \sum _{i=0}^{n-1} d_{i,i}\cdot a_{i,j}\cdot c_{i,j}.$ Now, from an encryption of *D*, we compute an encryption of *n*×*m* matrix *Expdiag*_0_(*D*) and then by rotating and summing

$$A\odot {\sf Expdiag}_{0}(D) \odot C = \left[ d_{i,i} \cdot a_{i,j}\cdot c_{i,j} \right]_{i,j}$$

through all rows, we obtain a matrix consisting of identical rows diag(*A*^*T*^*DC*). One can easily check that Algorithm 8 with input *C*_*A*_ and C.Mult(*C*_diag(*D*)_,*C*_*C*_)) exactly performs this computation, and then we omit the explicit algorithm. Note that this can be directly applied for diag(*S*^*T*^*WS*) computation of step 5 of Algorithm 4.

Toward our goal diag(*A*^*T*^*BA*) with a full matrix *B*, we exploit the above diagonal-case method after decomposing *B* into diagonal matrices. Let *B*_*t*_ be a diagonal matrix with the diagonal diag_*t*_(*B*) for 0≤*t*≤*n*−1, then it holds that

$${\textsf{diag}}\left(A^{T}BA\right) = \sum\limits_{t=0}^{n-1} {\textsf{diag}}\left(A^{T} \cdot B_{t} \cdot \rho_{t}(A)\right).$$

Therefore, after obtaining encryptions of *Expdiag*_*t*_(*B*) and *ρ*_*t*_(*A*) from encryptions of *B* and *A*, we can directly apply the diagonal-case method on each diag(*A*^*T*^·*B*_*t*_·*ρ*_*t*_(*A*)) computation for 1≤*t*≤*n* and finally obtain the encryption of diag(*A*^*T*^*BA*).

Here we again remark that, since these methods use Algorithm 8, they also ruin the column-wise packing as we already pointed out. Hence after applying these methods, it would be hard to perform another matrix operation. Indeed, one can check that the diagonal extractions are required for step 5 of Algorithm 4, which is the last part of algorithm that uses matrix structure.

### Approximate computation of sigmoid

Since the sigmoid function *σ*(*x*)=1/(1+ exp(−*x*)) is not a polynomial, we exploit an approximate polynomial of the function to evaluate based on HE. Following the methodology of [8, 22], we used least square approximation method over the interval [−8,8]. The approximate polynomials *g*(*x*) of degree 7 is computed as

$$0.5+1.735\cdot\frac{x}{8}-4.194\cdot\left(\frac{x}{8}\right)^{3} +5.434\cdot\left(\frac{x}{8}\right)^{5}-2.507\cdot\left(\frac{x}{8}\right)^{7}. $$

The maximal error between *σ*(*x*) and *g*(*x*) is approximately 0.032.

### Inverse of real numbers

In step 7 of Algorithm 4, we need to compute the inverse of *d*_*i*_ for 1≤*i*≤*m*. To compute the inverse of real numbers, we exploit the Goldschmidt’s division algorithm [31], which outputs an approximate value of the inverse through iterative polynomial evaluations. Refer to [32] for more details of the algorithm.

## Results

In this section, we present the experimental results of our modified semi-parallel GWAS algorithm based on HEAAN with a publicly available library [5]. All experiments were implemented in C++ 11 standard, and performed on Linux with Intel Xeon CPU E5-2620 v4 at 2.10GHz processor with multi-threading (8 threads) turned on.

### Dataset description

We used a dataset of 245 samples where each sample contains a binary phenotype, 3 covariates (height, weight, age), and 25,484 SNP data provided by iDASH 2018 competition. The dataset is divided into two sets named by iDash_Test and iDash_Eval each composed of 245 samples containing common phenotype and 3 covariates but different number of SNPs; 10,643 and 14,841 SNPs, respectively. We used iDash_Test to set optimal parameters, and iDash_Eval was used to evaluate our algorithm in the competition. Note that the first column of the covariate matrix $X\in {\mathbb {Q}}^{n\times k}$ is a vector of which all the components are 1. Therefore, the parameters are (*n*,*m*,*k*)=(245,10643,4) for iDash_Test and (*n*,*m*,*k*)=(245,14841,4) for iDash_Eval.

### Experimental setting and parameter selection

We propose two HEAAN parameter sets achieving 128-bit or higher security for two experiments denoted by Exp I and Exp II in Table 1. The security levels of HEAAN parameter sets were estimated with Albrecht’s security estimator [25, 26] of which inputs are the ring dimension *N*, the modulus *Q*, the Hamming weight h of a secret polynomial, and the error distribution *χ*_err_. Note that since the modulus of the evaluation key *evk* is 2^2*L*^, the security of HEAAN is estimated with input (*N*,*Q*=2^2*L*^,h,*χ*_err_).

**Table 1:** Parameters for HEAAN, and running time of KeyGen,Enc and Dec

Exp	HE params				Time (sec)		
	log*N*	*L*	*p*	h	KeyGen	Enc	Dec
I	17	1300	50	56	157	68	0.28
II	17	1700	50	78	197	90	0.15

Exp I is a streamlined version operating Algorithm 4 only until step 5; that is, it does not perform the last division process. Note that Exp II that includes the division process (step 7 of Algorithm 4) naturally requires larger *L* than Exp I.

We first set the scaling parameter *p* to be sufficiently large so that errors derived from HEAAN do not effect on significant bits of plaintexts. The level parameter *L* is chosen by considering the fact that *p* levels are consumed for each homomorphic multiplication. See “Approximate homomorphic encryption scheme HEAAN” section for specific definitions of HEAAN parameters. Besides thoese HEAAN parameters, one also needs to select an appropriate constant *α*>0 in Algorithm 3. After experimenting on several values, we set *α*=8. Note that the choice of *α* merely depends on the size of *X*, but not on *S*.

### Experimental results and evaluation

**Fig. 1:**
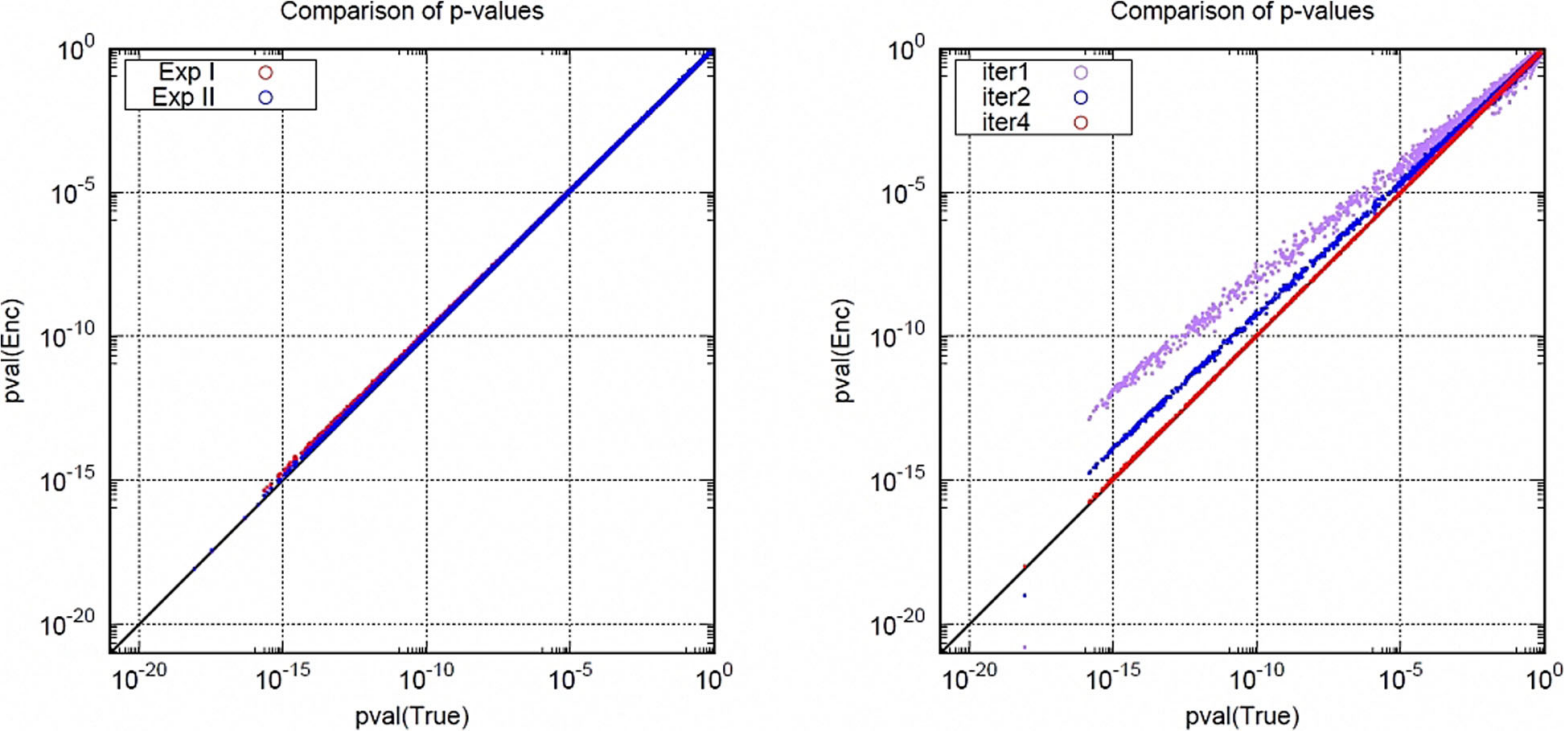
Comparison of *p*-values on iDASH datasets: **a** the left figure represents Exp I for iter=1,2,4 on iDash_Test, **b** the right figure represents Exp I and Exp II for iter=4 on iDash_Eval

We demonstrated our modified semi-parallel GWAS algorithm in encrypted state, and evaluated the accuracy of our algorithm comparing it to that of the original algorithm which is performed in unencrypted state. The comparison result of *p*-value is described as a (log-scale) graph in Fig. 1. We plotted each SNPs according to the *p*-values computed by original algorithm and ours denoted by True and Enc, respectively. The diagonal line represents the line *y*=*x*, and closer distribution of points to this line implies higher accuracy. The Fig. 1a shows that the accuracy of our algorithm increases with the number of iterations for Fisher scoring, where the data set iDash_Test is used. The Fig. 1b shows that the accuracy of Exp II, which includes a division procedure, is comparable to that of Exp I without the division, where the data set iDash_Eval is used. Comparing Exp I and Exp II, there exists a trade-off between computational time and information leakage. The output of Exp I is the vector of squared statistics (*z*_*i*_)_1≤*i*≤*m*_ which has exactly same information with the *p*-value vector pval, but it takes 20 minutes longer than Exp I. On the other hand, since Exp I outputs the numerator ${\textsf {det}}(U)\cdot c_{i}^{2}$ and the denominator *d*_*i*_ (in Algorithm 4) separately, it leaks some information more than *p*-values. However, it still seems to be very hard to extract any important information of input data from the numerator and denominator.

For more concrete evaluation, we classified each SNP as positive or negative depending on whether the corresponding *p*-value is larger or smaller than the given threshold (e.g. 10^−2^,10^−5^, or 10^−12^). Then, the accuracy of our algorithm compared to the original algorithm can be checked by a well-known statistical measure called *F*_1_ score. The *F*_1_ score of our algorithm is calculated by regarding the positive SNPs classified by the original GWAS algorithm to be the correct positive samples. For the formal definition of *F*_1_ score, we refer readers to [33].

The performance of our algorithm including the computation time and the *F*_1_ score on each parameter set is described in Table 2, where iter denotes the number of iterations in Fisher Scoring, Comp. time denotes the running time of our algorithm in encrypted state, and TH denotes the threshold of *p*-values for classification.

**Table 2:** Experimental results for each parameter set

Data	Params		Comp. time	Memory	*F*_1_ Score		
	Exp	iter		(GB)	TH: 10^−2^	TH: 10^−5^	TH: 10^−12^
iDash_Test	I	1	13 min*	9.3	0.960	0.969	0.243
	I	2	27 min	16.7	0.985	0.985	0.955
	**I**	**4**	**32 min**	**16.7**	**1.000**	**0.999**	**0.997**
	**II**	**4**	**52 min**	**22.0**	**1.000**	**0.999**	**0.998**
iDash_Eval	**I**	**4**	**38 min**	**19.4**	**0.998**	**0.995**	**0.992**
	**II**		**62 min**	**25.4**	**0.998**	**0.996**	**0.994**

*: We used more streamlined parameter; log*N*=16,*L*=950,*p*=50,h=91

As we have seen in Fig. 1, more iterations of Fisher scoring provides higher accuracy measured by higher *F*_1_ score. Note that 4 iterations suffice to provide high *F*_1_ score even in a very small threshold such as 10^−12^. Also, Exp II calculating approximate inverse in encrypted state provides almost similar *F*_1_ score to Exp I without such approximation. It implies that the error of inverse approximation does not seriously impact the whole approximation. Furthermore, Exp II shows even higher *F*_1_ score than Exp I due to the cancellation of errors from our algorithmic approximation and that from the inverse approximation.

For about 15,000 SNP data, our algorithm works in less than 40 minutes when we exclude step 7 of Algorithm 4 in encrypted state, or in about 60 minutes otherwise. We emphasize that each iteration of Fisher scoring takes about 3 minutes while the Goldschmidt’s division algorithm takes less than 30 seconds. Exp II takes much longer time than Exp I due to the larger level parameter *L*.

## Discussion

### Scalability

Our algorithm is executed and evaluated with about hundreds of samples each containing ten thousand SNPs, and 3 covariates which can be seen as a small-size data in usual GWAS analysis. We emphasize that our algorithm is highly scalable in the number of samples or SNPs, since we circumvent the naive execution of large-sized matrix operations through the proper algorithmic modification. To test the scalability of our algorithm in practice, we randomly generated 500 samples each of which consist of 3 covariates and 30,000 SNPs. Each column of the covariate matrix was uniform randomly generated in the interval [150,200],[40,100] and [20,80] considering height, weight and age, respectively. Each element of the SNP matrix was uniform randomly chosen as a binary matrix. The experiment Exp 1 on this random dataset encrypted with properly chosen hyperparameters iter=4 and *α*=2^−7^ still showed quite accurate *p*-value result compared to the result obtained by running Algorithm 2 in unencrypted state within 2 h.

### Fisher Scoring

Our HE-friendly modified Fisher scoring (Algorithm 3) works quite well in practice, but there still remains to obtain some theoretical results on the convergence of the algorithm with respect to the new parameter *α*. Furthermore, we should consider an error in every operation derived from HEAAN when homomorphically evaluate the algorithm. As a result, research on the convergence of the *erroneous* version of our modified Fisher scoring algorithm should be very interesting topic as a further work. In addition, we note that our modified Fisher scoring algorithm can be generally used for logistic regression, not restricted to GWAS algorithm.

## Conclusions

Interest on privacy-preserving genome data analysis based on HE has grown up very rapidly since the annual iDASH competition was launched, and GWAS is one of the most important technologies in this area which was also selected as one of three tasks in iDASH 2018 competition. Our HE-friendly modified semi-parallel GWAS algorithm was successfully implemented based on an approximate HE scheme HEAAN, and we could obtain the *p*-value result in about 30–40 minutes for 10,000–15,000 SNP data with sufficiently high accuracy compared to the result obtained in unencrypted state.

## Data Availability

The dataset was available to participants registered to iDASH 2018 competition.

## Abbreviations

HE: Homomorphic encryption
SNP: Single nucleotide polymorphism
GWAS: Genome-wide association study
TH: threshold

## References

[1] Malik MB, Ghazi MA, Ali R (2012). Privacy preserving data mining techniques: current scenario and future prospects. Third International Conference on Computer and Communication Technology (ICCCT).

[2] IDASH 2018. http://www.humangenomeprivacy.org/2018/. Accessed 15 Jan 2019.

[3] Cheon JH, Kim A, Kim M, Song Y (2017). Homomorphic encryption for arithmetic of approximate numbers. Advances in Cryptology–ASIACRYPT 2017: 23rd International Conference on the Theory and Application of Cryptology and Information Security.

[4] Cheon JH, Han K, Kim A, Kim M, Song Y (2018). Bootstrapping for approximate homomorphic encryption. Annual International Conference on the Theory and Applications of Cryptographic Techniques.

[5] Han K, Kim A, Kim M, Song Y. Implementation of HEAAN. https://github.com/snucrypto/HEAAN. Accessed 12 July 2018.

[6] Lauter K, López-Alt A, Naehrig M (2014). Private computation on encrypted genomic data. International Conference on Cryptology and Information Security in Latin America.

[7] Wang S, Zhang Y, Dai W, Lauter K, Kim M, Tang Y, Xiong H, Jiang X (2015). Healer: homomorphic computation of exact logistic regression for secure rare disease variants analysis in GWAS. Bioinformatics.

[8] Kim A, Song Y, Kim M, Lee K, Cheon JH (2018). Logistic regression model training based on the approximate homomorphic encryption. BMC Med Genet.

[9] Chen H, Gilad-Bachrach R, Han K, Huang Z, Jalali A, Laine K, Lauter K (2018). Logistic regression over encrypted data from fully homomorphic encryption. BMC Med Genet.

[10] Crawford JL, Gentry C, Halevi S, Platt D, Shoup V (2018). Doing real work with FHE: The case of logistic regression. Proceedings of the 6th Workshop on Encrypted Computing & Applied Homomorphic Cryptography.

[11] Bonte C, Vercauteren F (2018). Privacy-preserving logistic regression training. BMC Med Genet.

[12] IDASH 2017. http://www.humangenomeprivacy.org/2017/. Accessed 15 Jan 2019.

[13] Lu W, Yamada Y, Sakuma J (2015). Efficient secure outsourcing of genome-wide association studies. 2015 IEEE Security and Privacy Workshops.

[14] Bonte C, Makri E, Ardeshirdavani A, Simm J, Moreau Y, Vercauteren F (2018). Towards practical privacy-preserving genome-wide association study. BMC Bioinformatics.

[15] Jagadeesh KA, Wu DJ, Birgmeier JA, Boneh D, Bejerano G (2017). Deriving genomic diagnoses without revealing patient genomes. Science.

[16] Cho H, Wu DJ, Berger B (2018). Secure genome-wide association analysis using multiparty computation. Nat Biotechnol.

[17] Kamm L, Bogdanov D, Laur S, Vilo J (2013). A new way to protect privacy in large-scale genome-wide association studies. Bioinformatics.

[18] Constable SD, Tang Y, Wang S, Jiang X, Chapin S (2015). Privacy-preserving GWAS analysis on federated genomic datasets. BMC Med Inform Decis Making.

[19] Bogdanov D, Kamm L, Laur S, Sokk V (2018). Implementation and evaluation of an algorithm for cryptographically private principal component analysis on genomic data. IEEE/ACM Trans Comput Biol Bioinforma.

[20] Chen F, Wang S, Jiang X, Ding S, Lu Y, Kim J, Sahinalp SC, Shimizu C, Burns JC, Wright VJ (2016). Princess: Privacy-protecting rare disease international network collaboration via encryption through software guard extensions. Bioinformatics.

[21] Anati I, Gueron S, Johnson S, Scarlata V (2013). Innovative technology for cpu based attestation and sealing. Proceedings of the 2nd International Workshop on Hardware and Architectural Support for Security and Privacy vol. 13.

[22] Kim M, Song Y, Wang S, Xia Y, Jiang X (2018). Secure logistic regression based on homomorphic encryption: Design and evaluation. JMIR Med Inform.

[23] Cheon JH, Kim D, Kim Y, Song Y (2018). Ensemble method for privacy-preserving logistic regression based on homomorphic encryption. IEEE Access.

[24] Cheon JH, Han K, Hong SM, Kim HJ, Kim J, Kim S, Seo H, Shim H, Song Y (2018). Toward a secure drone system: Flying with real-time homomorphic authenticated encryption. IEEE Access.

[25] Albrecht MR, Player R, Scott S (2015). On the concrete hardness of learning with errors. J Math Cryptol.

[26] Albrecht MR. A sage module for estimating the concrete security of learning with errors instances. https://bitbucket.org/malb/lwe-estimator. Accessed 15 July 2018.

[27] Sikorska K, Lesaffre E, Groenen PF, Eilers PH (2013). Gwas on your notebook: fast semi-parallel linear and logistic regression for genome-wide association studies. BMC Bioinformatics.

[28] Longford NT (1987). A fast scoring algorithm for maximum likelihood estimation in unbalanced mixed models with nested random effects. Biometrika.

[29] Ruder S. An overview of gradient descent optimization algorithms. arXiv preprint arXiv:1609.04747. 2016.

[30] Juvekar C, Vaikuntanathan V, Chandrakasan A (2018). Gazelle: A low latency framework for secure neural network inference. 27th USENIX Security Symposium (USENIX Security 18).

[31] Goldschmidt RE. Applications of division by convergence. PhD thesis, Massachusetts Institute of Technology. 1964.

[32] Markstein P (2004). Software division and square root using goldschmidt’s algorithms. Proc 6th Conf Real Numbers Comput (RNC’6).

[33] Chinchor N (1992). Muc-4 evaluation metrics. Proceedings of the 4th Conference on Message Understanding.

